# Simultaneous induction of apoptosis and necroptosis by Tanshinone IIA in human hepatocellular carcinoma HepG2 cells

**DOI:** 10.1038/cddiscovery.2016.65

**Published:** 2016-10-03

**Authors:** C-Y Lin, T-W Chang, W-H Hsieh, M-C Hung, I-H Lin, S-C Lai, Y-J Tzeng

**Affiliations:** 1 Institute of Medical Sciences, Tzu Chi University, Hualien, Taiwan; 2 Division of Crop Improvement, Hualien District Agricultural Research and Extension Station, Council of Agriculture, Hualien, Taiwan; 3 Department of Public Health, Tzu Chi University, Hualien, Taiwan; 4 Department of Medical Imaging and Radiological Sciences, Tzu Chi University of Science and Technology, Hualien, Taiwan; 5 School of Post-Baccalaureate Chinese Medicine, Tzu Chi University, Hualien, Taiwan; 6 Department of Chinese Medicine, Buddhist Hualien Tzu Chi General Hospital, Hualien, Taiwan; 7 Department of Pharmacy, Buddhist Hualien Tzu Chi General Hospital, Hualien, Taiwan; 8 Department of Molecular Biology and Human Genetics, Tzu Chi University, Hualien, Taiwan; 9 Department of Life Science, Tzu Chi University, Hualien, Taiwan

## Abstract

Tanshinone IIA (Tan IIA), a constituent of the traditional medicinal plant *Salvia miltiorrhiza* BUNGE, has been reported to possess anticancer activity through induction of apoptosis in many cancer cells. Surprisingly, the present study finds that Tan IIA simultaneously causes apoptosis and necroptosis in human hepatocellular carcinoma HepG2 cells. We further find that apoptosis can be converted to necroptosis by pan-caspase inhibitor Z-VAD-fmk, and the two death modes can be blocked by necroptotic inhibitor necrostatin-1. The underlying mechanisms are revealed by analysis of the signaling molecules using western blotting. In control cells, FLICE inhibitory protein in short form (FLIP_S_) is expressed in relatively high levels and binds to caspase 8 in ripoptosome, which supposedly sustains cell survival. However, in Tan IIA-treated cells, FLIP_S_ is down-regulated and may thus cause homodimer formation of cleaved caspase 8, cleavage of receptor-interacting serine/threonine-protein kinases 1, 3 (RIP1, RIP3), and mixed-lineage kinase domain-like (MLKL), in turn leads to cell apoptosis. In parallel, Tan IIA causes necroptosis by forming a suggested necrosomal complex composed of RIP1/RIP3. Regarding the inhibitors, z-VAD-fmk diminishes the cleaved caspase 8, RIP1, RIP3, and MLKL induced by Tan IIA, and reconstructs the ripoptosome complex, which marks cells moving from apoptosis to necroptosis. Nec-1 recovers the Tan IIA down-regulated FLIP_S_, consequently causes FLIP_S_ to form heterodimer with caspase 8 and thus block apoptosis. Meanwhile, cleaved forms of RIP1 and RIP3 were observed preventing necroptosis. Intriguingly, the cytotoxicity of tumor necrosis factor-related apoptosis-inducing ligand to HepG2 cells is enhanced by Tan IIA in a pilot study, which may be attributed to low FLIP_S_ levels induced by Tan IIA. In short, Tan IIA simultaneously induces both Nec-1 inhibition and FLIP_S_ regulation-mediated apoptosis/necroptosis, which has not been previously documented. Moreover, the involvement of the cleavage type of MLKL in executing necroptosis warrants further investigation.

## Introduction

Apoptosis, a kind of cell death, is a natural way to prevent the development of cancer. Thus, determination of the apoptosis-inducing capability has emerged as a mainstream approach for qualifying anticancer agents. Nevertheless, cancer cells can develop resistance to such agents by overcoming apoptosis, thus raising challenges to conventional therapies. Targeting cell death pathways other than apoptosis should provide a new direction for drug design or screening.

Necroptosis has been recently observed to be a form of programmed necrosis. It is mediated by a complex derived from an assembly of signaling molecules named ripoptosomes. The ripoptosome complex serves as a platform for determining cell survival, apoptosis, or necroptosis. Although some corresponding complexes vary in terms of initiator, modulator, or effecter components depending on different cell types,^1^ the well-known composition of ripoptosome is caspase 8, Fas-associated death domain protein (FADD), and two receptor-interacting serine/threonine-protein kinases RIPK1 and RIPK3.^2^ Caspase 8 is an apoptosis effector, FADD is an adaptor, RIP1 and RIP3 are necroptotic effectors, and FLIP is a modulator. FLIP structurally resembles caspase 8 in which the proteolysis activity is lost by replacement of catalytically active cysteine with a tyrosine or multiple amino acids.^3,4^ FLIP is expressed as splice variants in humans, that is, long (FLIP_L_) and short (FLIP_S_). Both FLIP_L_ and FLIP_S_ can bind to caspase 8 with high affinity for exerting their regulator role. When FLIP is expressed at high levels, it forms a heterodimer with caspase 8 and thus inhibits its homodimer formation, consequently blocking apoptosis and preventing necrosis by inactivating RIP3, thus causing cells survival. However, low levels of FLIP bifurcate the cell fate into caspase 8-dependent apoptosis and RIP3-dependent necroptosis, which is determined by FLIP binding. When the caspase 8 of the ripoptosome is free of FLIP binding, it becomes an active form of the homodimer through auto-proteolysis, and triggers the downstream signaling of apoptosis, such as caspase 3. Meanwhile, its neighbor components RIP1 and RIP3 are cleaved, leading to the formation of an apoptotic ripoptosome but they fail to perform necroptosis. On the other hand, in the absence of FLIP, the RIP1/ RIP3 complex (i.e., a necrosome) dissociates from the ripoptosome and makes necroptosis available.^5,6^ RIP3 of the necrosome is phosphorylated and in turn recruits mixed-lineage kinase domain-like (MLKL) and phosphorylate it, resulting in it being oligomerized, translocated to plasma membrane and eventually forming a calcium influx-mediated pore.^7^

Tanshinone IIA (Tan IIA), a component isolated from the roots of *Salvia miltiorrhiza* BUNGE, is an herbal medicine used in East Asia to treat cardiovascular diseases. Tan IIA has been documented to exhibit anti-angiogenic, anti-oxidant, anti-inflammatory and apoptotic properties. As described in our previous report,^8^ Tan IIA has been characterized for anticancer activity in various solid tumor cells in the prostate, liver, bone, oral cavity, esophagus, and cervix; it has also been found to be active against chronic myeloid leukemia cells. Like most cancericidal phytochemicals, the cytotoxic activity of Tan IIA has caused apoptosis in gastric, colon, breast, ovarian, lung, and leukocytic cancer cell lines. Recently, the herbal extract component neoalbaconol has been reported to induce necroptosis in cell lines of human amelanotic melanoma (A375), human breast cancer (MX-1), human gastric cancer (AGS-EBV), and the mouse fibrosarcoma (L929).^9^ In addition, shikonin stimulates both apoptosis and necroptosis in HL60 and K562 leukemia cells.^10^ However, whether Tan IIA triggers necroptosis is yet to be investigated.

This study is the first to indicate that Tan IIA kills HepG2 cells simultaneously through apoptosis as well as necropoptosis. Furthermore, we reveal the respective influences of apoptosis inhibitor z-VAD-fmk and necroptosis inhibitor Nec-1, and suggest the underlying signaling mechanisms for the findings on the basis of ripoptosome and necrosome.

## Results

### Tan IIA induces both apoptosis and necroptosis in HepG2 cells

**Figure 1:**
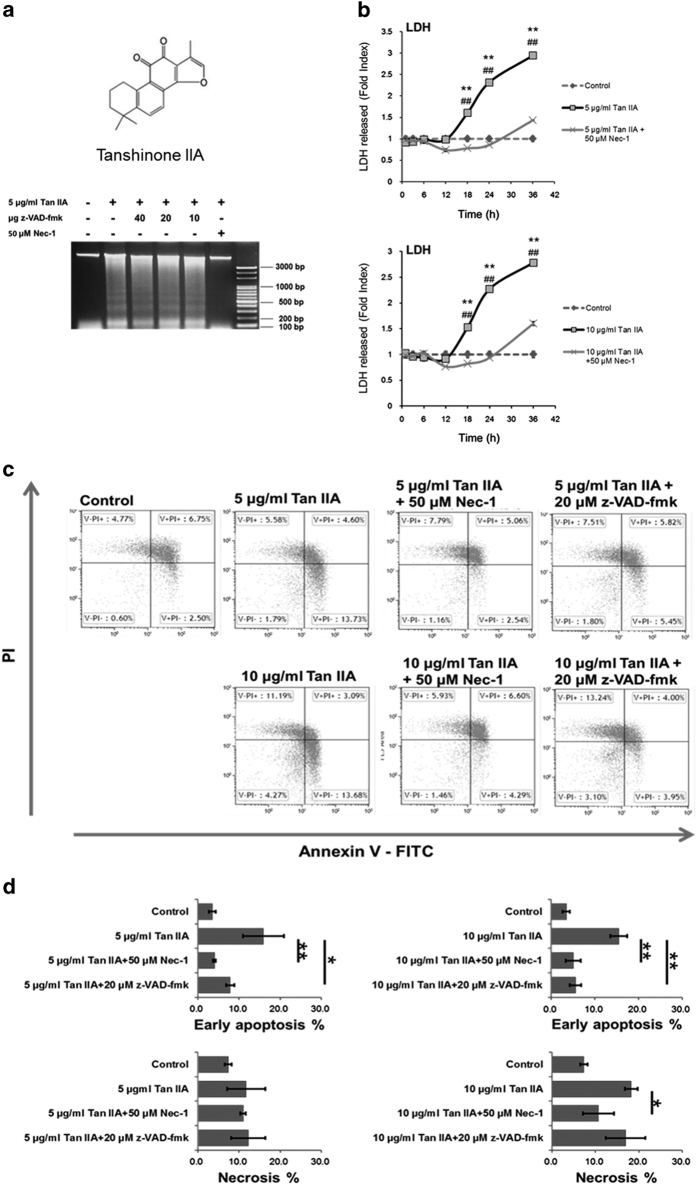
Apoptotic and necroptotic response of HepG2 cells treatment with Tan IIA alone or combined treatment with z-VAD-fmk and Nec-1, respectively. (**a**) Chemical structure of Tan IIA and DNA fragmentation. HepG2 cells were pretreated with 20 *μ*M z-VAD-fmk or 50 *μ*M Nec-1 for 30 min, and subsequently treated with 5 or 10 *μ*g/ml Tan IIA for 12 h. DNA is isolated from the cells and separated on agarose gel to show the patterns of DNA fragmentation as smears and nucleosomal ladders induced by Tan IIA. The two patterns were removed by Nec-1. (**b**) HepG2 cells were pretreated with 20 *μ*M z-VAD-fmk or 50 *μ*M Nec-1 for 30 min, and subsequently treated with 5 or 10 *μ*g/ml Tan IIA for 1-36 h. Time-dependent LDH activity elicited by Tan IIA, is Nec-1-inhibitable. Data are presented as mean±S.D.; *N*=4 independent experiments. Statistical analysis was carried out using Student’s *t*-test (paired, one-tailed, **P<0.01 Tan IIA treatment *versus* control, ^##^*P*<0.01 Tan IIA *versus* Tan IIA/Nec-1 treatment). (**c**, **d**) HepG2 cells were pretreated with 20 *μ*M z-VAD-fmk or 50 *μ*M Nec-1 for 30 min, and subsequently treated with 5 or 10 *μ*g/ml Tan IIA for 12 h. (**c**) Histograms shows the portions of cells undergoing early apoptosis or necroptosis. Cell death was measured after staining with Annexin V-FITC and propidium iodide (PI) followed by flow cytometry. (**d**) Quantitative analysis of early apoptosis and necroptosis cells in percentage. z-VAD-fmk rescues the cells from the apoptosis induced by Tan IIA, whereas Nec-1 rescues from apoptosis and necroptosis. Data are presented as mean±S.D.; *N*=3 independent experiments. Statistical analysis was carried out using Student’s *t*-test (paired, one-tailed, *P<0.05, **P<0.01).

**Figure 2:**
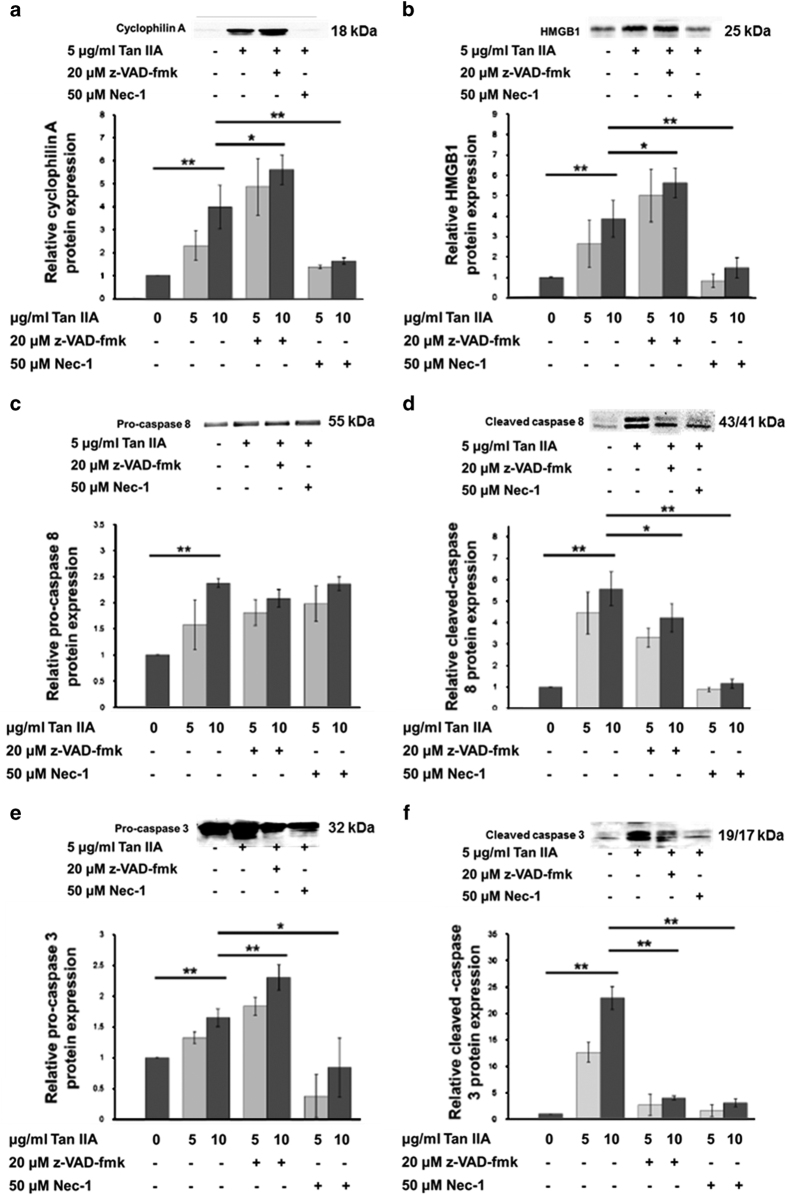
z-VAD-fmk inhibits expression levels of apoptotic markers but enhances necroptotic markers; however, Nec-1 inhibits both apoptotic and necroptotic markers. HepG2 cells were pretreated with 20 *μ*M z-VAD-fmk or 50 *μ*M Nec-1 for 30 min, and subsequently treated with 5 or 10 *μ*g/ml Tan IIA for 12 h. Protein from cytoplasmic extracts of HepG2 cells followed by western blot analysis. Quantitative data are presented as mean±S.D.; *N*=3 independent experiments. Statistical analysis was carried out using Student’s *t*-test (paired, one-tailed, *P<0.05, **P<0.01). (**a**, **b**) Expression levels of necropoptic markers cyclophilin A and HMGB1 were elevated by Tan IIA, and were re-elevated by z-VAD-fmk but repressed by Nec-1. (**c**) Expression levels of apoptotic marker pro-caspase 8 were elevated by Tan IIA. (**d**) Cleaved form of caspase 8 was elevated by Tan IIA, but repressed by both z-VAD-fmk and Nec-1. (**e**) Expression levels of apoptotic marker pro-caspase 3 were elevated by Tan IIA and z-VAD-fmk but repressed by Nec-1. (**f**) Cleaved form of caspase 3 was elevated by Tan IIA, but repressed by both z-VAD-fmk and Nec-1.

Cytotoxicity and apoptosis induced by Tan IIA in HepG2 cells has been well characterized.^11,12^ However, our previous and present results found two patterns of DNA fragmentation, a nucelosomal and a smear (Figure 1a),^8^ respectively, representing apoptosis and necrosis.^13^ We intend to confirm this finding by means of Annexin V and FITC, and flow cytometry analysis. The results showed that early apoptotic (Annexin V+PI−) and necrotic (Annexin V-PI+) cells were detectable (Figures 1c and d). Necroptosis can be confirmed by measuring the activity of lactate dehydrogenase (LDH) released from cells.^14^ With this method we determined that Tan IIA-induced necroptosis in a time-dependent manner, which was Nec-1-inhibitable (Figure 1b). Next, western blot analysis was subjected to detect the necroptotic markers cyclophilin A and HMGB1. Results indicated that the two markers were evoked by Tan IIA (Figure 2). These results indicate that Tan IIA simultaneously causes apoptosis and necroptosis in HepG2 cells.

### z-VAD-fmk converts apoptosis to necroptosis in Tan IIA-treated HepG2 cells

**Figure 3:**
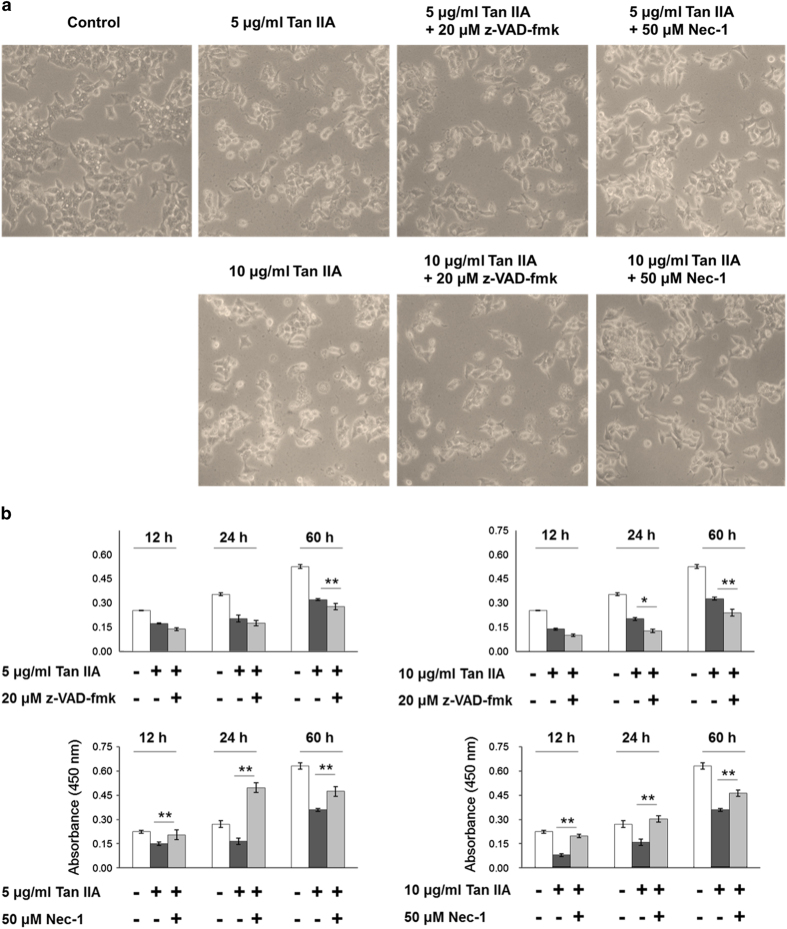
Nec-1 rather than z-VAD-fmk influenced by Tan IIA recovers the cell shape and viability. (**a**) HepG2 cells were pretreated with 20 *μ*M z-VAD-fmk or 50 *μ*M Nec-1 for 30 min, and subsequently treated with 5 or 10 *μ*g/ml Tan IIA for 12 h. Microscopic images show that Tan IIA treatment results in reverting the rounding-up of cell shape reverts to polygonal as control cells by Nec-1 but not by z-VAD-fmk. (**b**) HepG2 cells were pretreated with 20 *μ*M z-VAD-fmk or 50 *μ*M Nec-1 for 30 min, and subsequently treated with 5 or 10 *μ*g/ml Tan IIA for 12, 24, and 60 h. Cell viability was measured using the WST-1 assay and presented as absorbance values at 450 nm. Quantitative data are presented as mean±S.D.; *N*=4 independent experiments. Statistical analysis was carried out using Student’s *t*-test (paired, one-tailed, **P<0.01).

**Figure 4:**
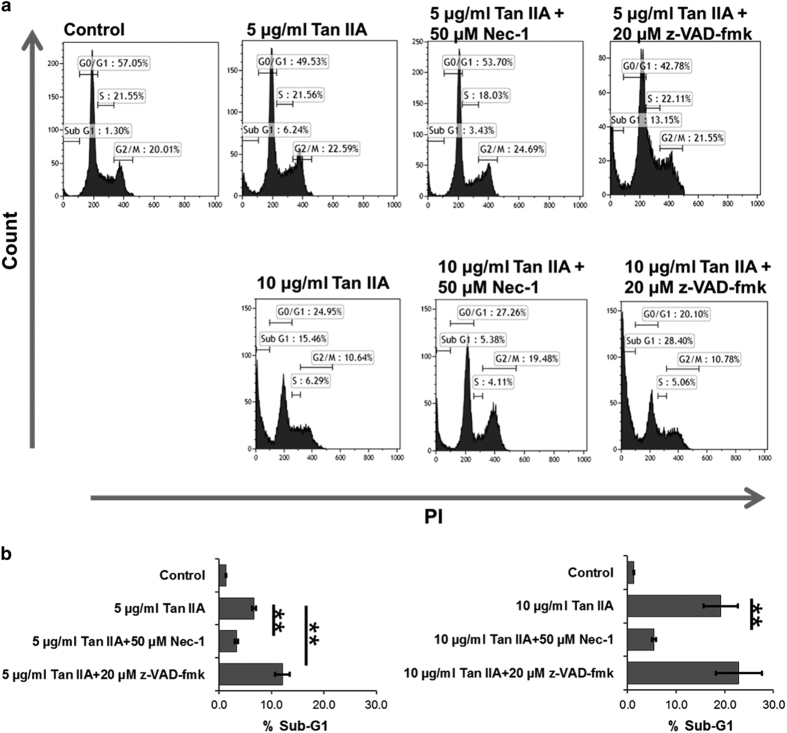
Nec-1 rather than z-VAD-fmk rescues the Sub-G1 portions of induced by Tan IIA. HepG2 cells were pretreated with 20 *μ*M z-VAD-fmk or 50 *μ*M Nec-1 for 30 min and subsequently treated with 5 or 10 *μ*g/ml of Tan IIA for 12 h. Sub-G1 portions were identified by flow cytometry using PI staining. (**a**) Histograms show Sub-G1 portions on various treatments. (**b**) Quantitative data of the Sub-G1 portions are presented as mean±S.D.; *N*=3 independent experiments. Statistical analysis was carried out using Student’s *t*-test (paired, one-tailed, **P<0.01).

**Figure 5:**
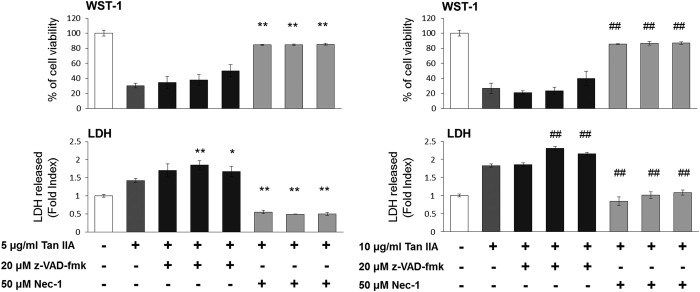
Various levels of Tan IIA reduces cell viability and is sustained by z-VAD-fmk but rescued by Nec-1, whereas necroptotsis is increased by z-VAD-fmk but decreased by Nec-1. HepG2 cells were pretreated with various concentrations of z-VAD-fmk or Nec-1 for 30 mins and then with Tan IIA for 12 h. Cell viability was measured using WST-1 assay and necroptosis using LDH release assay. Data are presented as mean±S.D.; *N*=4 independent experiments. Statistical analysis was carried out using Student’s *t*-test (paired, one-tailed, **P<0.01, ^##^*P*<0.01 for compared with 5  and 10 *μ*g/ml of Tan IIA, respectively).

Most of HepG2 cells displayed a polygonal cell shape, whereas they were occasionally found to be rounding-up after treatment with Tan IIA. Furthermore, z-VAD-fmk could not revert the shape of cells from rounding-up to polygonal (Figure 3a). Consistently from the results of WST-1 assay, cell survival rates induced by Tan IIA could not be recovered by z-VAD-fmk (Figure 3b). Similar results were obtained from observation on the cells treated with Tan IIA and subsequently analyzed by PI staining/flow cytometry, the sub-G1 portions were not rescued by z-VAD-fmk (Figure 4) either. In fact, z-VAD-fmk is not functionless, as it indeed inhibited Annexin V defined apoptosis evoked by Tan IIA (Figures 1c and d). Nevertheless, portions of the sub-G1 phase were significantly elevated by co-treatment of 5 *μ*g/ml of Tan IIA and 20 *μ*M with z-VAD-fmk (Figure 4), raising the possibility that inhibition of apoptosis results in increasing necroptosis. This suggestion was supported by elevated expression levels of necroptotic markers cyclophilin A and HMGB1 observed in the cells treated with Tan IIA/z-VAD-fmk (Figure 2a and b) and further by increased levels of necroptosis with LDH assay (Figure 5). This suggests that z-VAD-fmk converts Tan IIA-induced apoptosis to necroptosis, explaining why cell proliferation rates sustain even after the treatment with z-VAD-fmk.

### Nec-1 abrogates both the apoptosis and the necroptosis induced by Tan IIA

In contrast to z-VAD-fmk, Nec-1 rescued the cell shape from the Tan IIA-induced rounding-up to polygonal (Figure 3a). We observed that necrosis inhibitor Nec-1 eliminated both the smear and the necleosomal DNA fragmentation induced by Tan IIA (Figure 1a). Consistently, we found that, apoptotic and necrotic cell populations were inhibited by Nec-1, which was analyzed with Annexin V/FITC staining (Figures 1c and d). We thereafter determined that Tan IIA-induced LDH activity was diminished by Nec-1 (Figure 1b), and the expression levels of necrotic markers cyclophilin A and HMGB1 were likewise significantly reduced by Nec-1 (Figure 2a and b). Moreover, it reduced the sub-G1 portions stimulated by Tan IIA (Figure 4). From the results above, we conclude that Nec-1 eliminates both apoptosis and necroptosis induced by Tan IIA.

### FLIP plays a central role in regulating the cell death induced by Tan IIA

**Figure 6:**
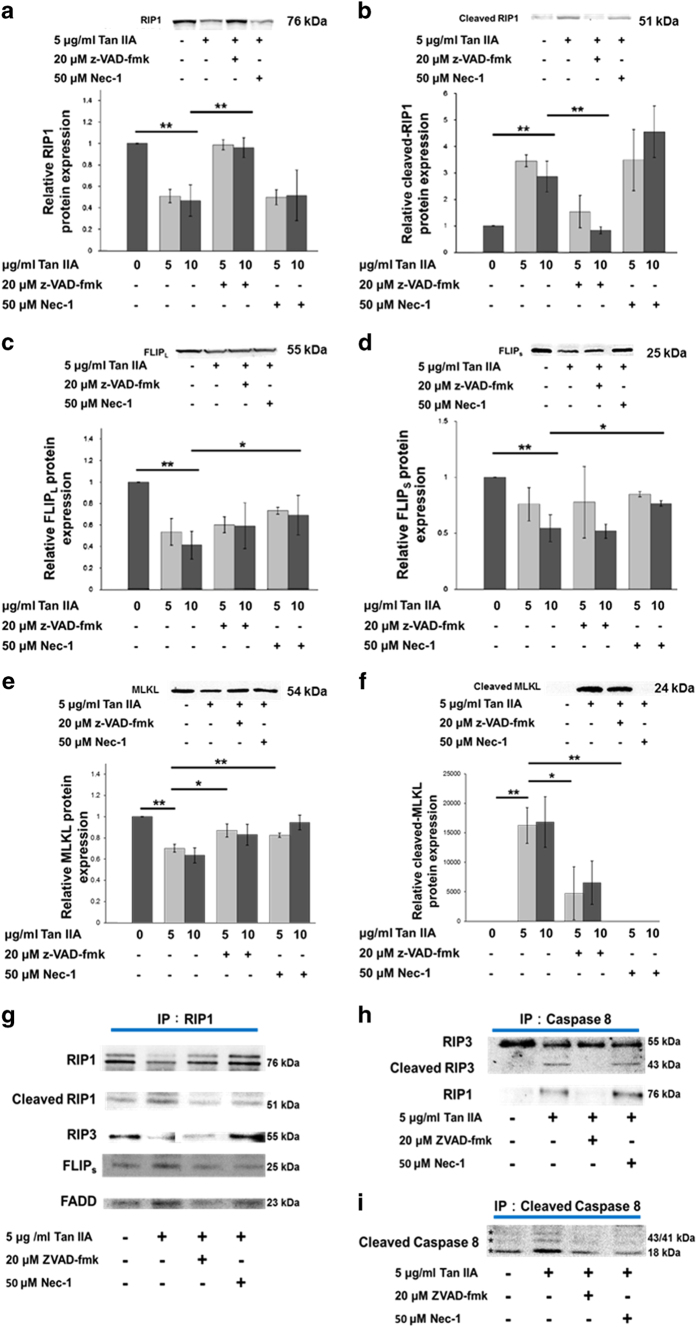
Influences of z-VAD-fmk and Nec-1 on apoptotic and necroptotic signaling proteins, respectively. HepG2 cells were pretreated with 20 *μ*M z-VAD-fmk or 50 *μ*M Nec-1 for 30 min and subsequently treated with 5 or 10 *μ*g/ml of Tan IIA for 12 h. (**a**–**f**) Cell lysates were analyzed by western blot with indicated antibodies. Quantitative data are presented as mean±S.D.; *N*=3 independent experiments. Statistical analysis was carried out using Student’s *t*-test (paired, one-tailed, *P<0.05, **P<0.01). (**a**) Expression levels of RIP1 in intact form were reduced by Tan IIA, and recovered by co-treatment with z-VAD-fmk but not with Nec-1. (**b**) Expression levels of RIP1 in cleaved form were increased by Tan IIA, and reduced by co-treatment with z-VAD-fmk but not with Nec-1. (**c**, **d**) Expression levels of FLIP_L_ and FLIP_S_ were reduced by Tan IIA but recovered by co-treatment with Nec-1. (**e**) Expression levels of MLKL in monomer form were reduced by Tan IIA but recovered by co-treatment with both z-VAD-fmk and Nec-1. (**f**) Poor levels of MLKL expression in control cells were elevated by Tan IIA but reduced by co-treatment with z-VAD-fmk and Nec-1. (**g**) Anti-RIP1 pAb IP samples were analyzed by anti-RIP1 pAb, anti-RIP3 pAb, anti-FLIPS pAb, anti-FADD pAb western blot. (**h**) Anti-caspase 8 pAb IP samples were analyzed by anti-RIP1 pAb, anti-RIP3 pAb Western blot. (**i**) Anti-cleaved caspase 8 pAb IP samples were analyzed by Anti-cleaved caspase 8 pAb western blot.

We analyzed the signaling pathway responsible for cell survival, apoptosis, and necroptosis by means of western blotting and IP western blotting. In the control cells, we found that FLIP_L_, FLIPs, pro-caspase 8, RIP1, and RIP3, were demonstrable (Figures 6a–d). We further investigated the assembly of these molecules by means of IP western blotting. The results showed that RIP1 could aggregate with RIP3, FADD, pro-caspase 8, and FLIPs instead of FLIP_L._ We suggest that the molecules assemble as a ripoptosome-like survival complex RIP1/RIP3/FADD/FLIP_S_/caspase 8. In addition, small amount of cleaved RIP1 was detected, which might be processed by caspase 8 after forming a complex with FLIP.^15–19^

On treatment with Tan IIA, both FLIP_L_ and FLIPs were down-regulated (Figures 6c and d). The levels of intact RIP1 were decreased, whereas those of cleaved RIP1 were increased (Figures 6a and b). Moreover, cleaved caspase 8 and caspase 3 (i.e., active forms) were predominant in the western blotting (Figure 2d and f), and homodimer of cleaved caspase 8 was found in IP western blot analysis (Figure 6i). These results imply that low levels of the two FLIP isoforms result not only in the activation of caspase 8 and caspase 3, but also the cleavage of RIP1, a typical apoptotic situation. Regarding their assembly, an aggregation of lower levels of RIP1 and RIP3, along with higher levels of cleaved RIP1, FLIPs, and FADD were found in Tan IIA-treated cells, in comparison with control cells by means of the IP western blotting strategy (Figure 6g). Further results confirmed that pro-caspase 8 can form death complexes with RIP1, RIP3 and the cleaved form of RIP3 (Figure 6h). Moreover, FLIP_L_ was not as obvious as FLIP_S_ in complex formation (data not shown). It seems that some portion of the survival complex was changed by Tan IIA to become apoptotic in which caspase 8 was active and further cleaved RIP1 and RIP3 to result in apoptosis presumably in some cells.^20–22^ At the same time, in other cells the formation of a necrotic complex RIP1/RIP3 was suggested on the basis of previous reports.^23^

Moreover, the apoptotic complex was confirmed by the administration of a pan-caspase inhibitor z-VAD-fmk into Tan IIA-treated cells. Results showed that the elevated levels of cleaved RIP1 were decreased to the control levels (Figure 6a) and the cleaved form of RIP3 (Figure 6b), caspase 8 (Figures 2c and d) and caspase 3 (Figures 2e and f) were separately eliminated by the inhibitor. As inhibition of apoptosis could results in increased necroptosis (Figures 1c and d, 2a–f and 5). Tan IIA/z-VAD-fmk treated cells should display the necroptotic complex based on the IP western blotting results. From the western blotting results (Figure 6g), we proposed a complex of RIP1/RIP3 as speculated above and observed a presumable trace of ripoptosome.

We further added the necroptosis inhibitor Nec-1 into Tan IIA-treated cells and discovered that the expression levels of FLIP_L_ and FLIP_S_ were increased in comparison with Tan IIA treatment alone. In addition, the cleaved forms of caspase 8/3 were substituted by intact forms (Figures 2c–f). This can be used to illustrate how Nec-1 can inhibit Tan IIA-induced caspase-dependent apoptosis. Meanwhile, RIP1 and RIP3 were cleaved more prominently in comparison with the control (Figures 6a and b), and might thus lose activity and eventually block necrosis.

Conclusively, Tan IIA-induced apoptosis/necroptosis may be mediated with ripoptosome and necrosome, which are associated with expression levels of FLIP and processing of RIP1 and RIP3.

### Cleavage/intact forms of MLKL are, respectively, associated with apoptosis/necroptosis

MLKL protein has been reported to be involved in necroptosis by forming oligomers on plasma membrane.^7^ We thus at first determined its monomer type by using western blot analysis and found that it was down-regulated by Tan IIA and rescued by z-VAD-fmk and Nec-1 (Figure 6e). Similar results were determined on the trimmer type (Supplementary Information). These results indicated the poor association of both forms of MLKL with necroptosis. On the other hand, we found that a cleaved type of MLKL was prominent in the cells treated with Tan IIA, but not in the negative control. The levels of the cleavage form were recovered from cleaved to intact by z-VAD-fmk and Nec-1 treatment, respectively (Figure 6f). The former is partially but significantly recovered by z-VAD-fmk, whereas the latter is totally recovered by Nec-1. It is thus suggested that MLKL is cleaved by caspase on apoptosis, but maintains intact on necroptosis. The intact form might further be phosphorylated by RIP3, resulting in necroptotic membrane rupture. Altogether, these results demonstrate that MLKL is processed by caspase 8 and links to cell apoptosis and necroptosis induced by Tan IIA.

### Tan IIA synergistically enhances cytotoxicity of TRAIL to HepG2 cells

**Figure 7:**
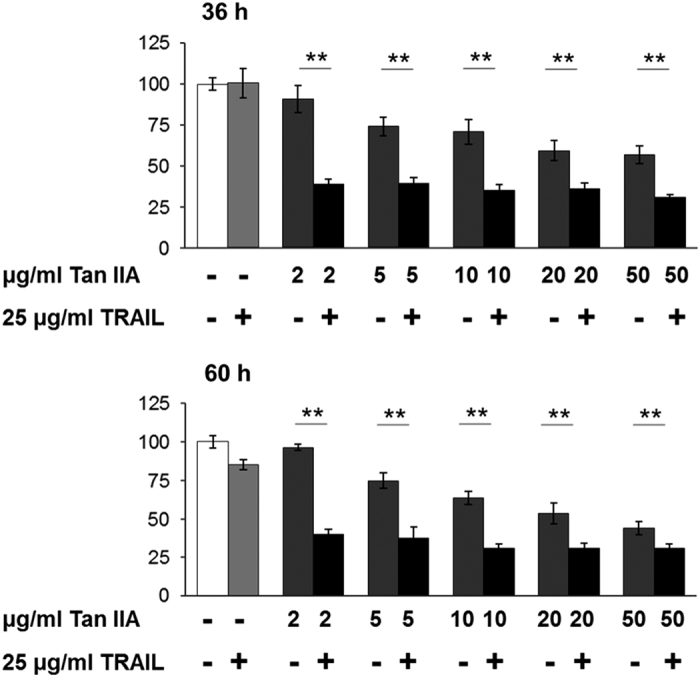
Combination of Tan IIA and TRAIL enhances cytotoxicity. HepG2 cells were treated with 25 *μ*g/ml of TRAIL in combination with various concentrations of Tan IIA as indicated. Cell viability was measured with WST-1 assay and quantitative data are presented as mean±S.D.; *N*=4 independent experiments. Statistical analysis was carried out using Student’s *t*-test (paired, one-tailed, **P<0.01).

As described above, the FLIP has a regulatory role in Tan IIA-induced cell death, on this basis down-regulation of FLIP should lead to cell death. TRAIL has been known to induce apoptosis through binding with DISC, which in turn recruits and activates pro-caspase 8 proteolytically to initiate an apoptosis cascade. It has been reported that down-regulation of FLIP_L_ and FLIP_S_ enhances caspase 8 recruitment by activation at the DISC.^24^ As our results indicate that Tan IIA reduces the expression levels of FLIP_L_ and FLIP_S_, it should provide a synergistic benefit to enhance TRAIL cytotoxicity through causing an increased rate of apoptosis in addition to necroptosis. As expected, combinative treatments of Tan IIA and TRAIL significantly reduced cell survival rates (Figure 7).

## Discussion

**Figure 8:**
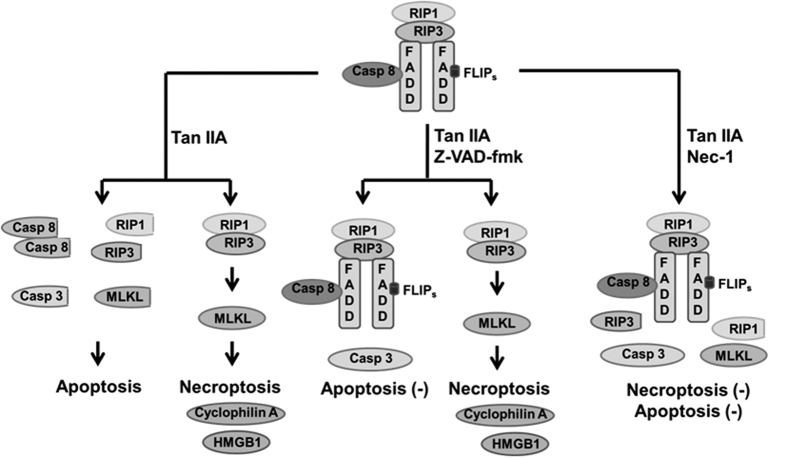
Proposed model for tanshinone IIA (Tan IIA) simultaneously inducing both apoptosis and necroptosis, and regulated by FLIP-associated pathway. In HepG2 cells, FLIP_S_ binds to ripotosomes, which hampers the auto-proteolysis of caspase 8 and blocks apoptosis. In the meantime, the FADD bound RIP1/RIP3 is inactive and thus fails to cause necroptosis. When the cells were treated with Tan IIA, FLIP is down-regulated, leading to dissociation of ripotosome, auto-proteolysis of caspase 8, cleavage of caspase 3, RIP1, RIP3, and MLKL, and consequently causing some cells to undergo apoptosis. Meanwhile, Tan IIA also induces necrosis in other sets of cells by forming a necrosome composed of RIP1/RIP3 heterodimer and mediating a necrotic executor MLKL. z-VAD-fmk inhibits apoptosis, rather than necroptosis, by blocking the auto-proteolysis of caspase 8. Nec-1 recovers the FLIP down-regulated by Tan IIA, which results in FLIPS binding to ripotosome and blocking apoptosis. On the other hand, RIP1 and RIP3 both remain cleaved, although MLKL reverts into intact, resulting in lack of necroptosis.

This study shows that Tan IIA causes apoptosis and necroptosis simultaneously in HepG2 cells. z-VAD-fmk converts the apoptosis to necroptosis, and both modes of cell death can be inhibited by Nec-1. FLIP_S_ has a central role in regulating the two modes of cell death. In addition, cleavage of RIP1, RIP3 and MLKL is associated with the regulation. A schematic overview of major signal transduction pathways dealing with these findings is shown in Figure 8 of this article.

Necroptosis was initially identified as a backup and alternative to apoptosis; that is, it is incompatible with apoptosis.^25^ This is evidenced by the emergence of necroptosis on treating L929 cells with apoptosis inhibitor z-VAD-fmk,^26^ and Nec-1 was reported to revert shikonin-induced necroptosis to apoptosis.^10^ Surprisingly, we found that Tan IIA can induce death of HepG2 cells through apoptosis and necroptosis. It seems that these two modes occur in separate cells, as the insight mechanisms are mutually exclusive. This raises an open question about how Tan IIA treatment bifurcates the cells into apoptotic or necroptotic groups.

In the present study, co-treatment of Tan IIA/Nec-1 results in processing of RIP1 and RIP3. This is incomprehensible, as in this situation, caspase 8 and caspase 3 are prominent as inactive forms, and thus lack sufficient activity to cleave RIP1 and RIP3. Nevertheless, it has been found that the heterocomplex of FLIP_L_/caspase 8 is proteolytically active and cleaves local substrates such as RIPs.^15^ Moreover, FLIP_L_ has been reported to induce the activity of its partner, caspase 8, without cleaving its interdomain and to change its substrate specificity.^19^ Although both FLIP_L_ and FLIP_S_ are expressed in Nec-1-treated cells, only FLIP_S_ was shown to be a component of ripoptosome. It is suggested to bind to FADD and thus inactivate caspase 8.

Although the proposed ripoptosome and necrosome have been previously suggested in the literature, our study raises several new aspects warranting further investigation. First, Nec-1, regarded as a specific necroptosis inhibitor, has been considered to be ineffective against apoptosis. However, we found that Nec-1 inhibits both necroptosis and apoptosis. As our data shows, Nec-1 sustains the necroptotic key factors RIP1 and RIP3 in cleaved form, and rescues the decreased levels of FLIP_L_/FLIP_S_ expression. The former supposedly blocks the triggering of MLKL-necroptosis signaling, and the latter may inhibit the activation of apoptosis-mediated enzyme pro-caspase 8. Second, the protein MLKL induces necroptosis by directly permeabilizing the plasma membrane, and is activated by phosphorylation by RIP3. Its cleaved form should result from the processing by caspase, and thus prevent cell death by necrosis. Moreover, we observed that Nec-1 can reduce cleavage due to the blocking of caspase signaling. However, this needs to be further studied and verified.

As shown in present study FLIP is down-regulated by Tan IIA and involved in cell death. Taking advantage of this finding, combination of Tan IIA and TRAIL has been found to exert synergistic cytotoxicity against HepG2 cells. Based on this, Tan IIA may be used as a key component to combine with other FLIP-regulable chemicals, of which the synergistic effect may be useful for cancer prevention. Moreover, this design may be especially useful for breaking through the drug resistance of cancer cells against TRAIL.

Taken together, the findings of Tan IIA-induced apoptosis and necroptosis in HepG2 cells offer new research directions for the inhibition mechanism of Nec-1 to apoptosis, the necroptotic role of MLKL cleavage, and the combinational cytotoxic enhancement of components with Tan IIA.

## Materials and methods

### Cell line and culture

The human hepatoma cell line HepG2 was kindly provided by Dr. SY Lo at Tzu Chi University. The cells were maintained in Dulbecco’s modified Eagle’s medium with phenol red (DMEM; Gibco, New York, USA) supplemented with 10% fetal bovine serum (FBS, Biological Industries, Kibbutz Beit Haemek, Israel), 100 U/ml penicillin and 100 *μ*g/ml streptomycin (Biological Industries, Kibbutz Beit Haemek, Israel), in a humidified incubator under 37 °C, 5% CO_2_ conditions.

### Chemical compounds

High-performance liquid chromatography (HPLC)-grade Tan IIA with a purity of 98% was purchased from Kesure Biotechnology Co. (Kunming, PRC). Necrostatin-1(Nec-1) with a purity of 99.9% was purchased from Enzo Life Sciences (New York, NY, USA). Nec-1-mediated inhibition necroptosis by inhibiting RIP1 activity. z-VAD-fmk with a purity of 99.9% was purchased from ApEXBio (Hsinchu, Taiwan). The agents were dissolved in DMSO (dimethyl sufoxide) at a concentration of 2 mg/ml Tan IIA, 20 mM z-VAD-fmk and 50 mM Nec-1 as stock.

### Cell viability assay

The WST-1 (4-[3-(4-iodophenyl)-2-(4-nitrophenyl)-2H-5- tetrazolio]-1,3-benzene disulfonate) cell proliferation and cytotoxicity assay (Roche Co., South San Francisco, CA, USA) was used to determine cell viability based on the reduction of tetrazolium salt to formazan by mitochondrial dehydrogenases. For the cell growth assay, 1×10^4^ exponentially growing cells were plated to each well of a 96-well tissue culture plate and incubated overnight in the same conditions as those described above to allow cells to attach to the bottom of each well. The cells were then treated with concentrations 5 or 10 μg/ml Tan IIA, combined Tan IIA and Nec-1 and combined Tan IIA and z-VAD-fmk for 12, 24 and 60 h. The negative control group was treated with an equal concentration of the solvent vehicle (DMSO). After exposure, the medium was removed and washed with 200 *μ*l PBS. A mixture of 10 *μ*l Cell Proliferation Reagent WST-1 and 190 *μ*l phenol red free DMEM was then added for 30 min under the same incubation conditions. The absorbance was measured in a microplate reader (Thermo Scientific Multiskan Spectrum, New York, NY, USA) at 450 nm. Microsoft Excel 2010 was used for all data entry and analysis. Data are presented as mean ±S.D. of the quadruplicate. Student's two-tailed *t*-test was used to analyze statistical significance between groups.

### Analysis of cell death by flow cytometry

Exponentially growing HepG2 cells were treated with Tan IIA, Nec-1 and z-VAD-fmk mixture at the desired concentrations, following removal of non-adherent cells by gentle washing. After exposure to the drugs for 12 h, the cells were collected and centrifuged at 500×*g* in a 15 ml tube for 5 min. The cells were then washed with ice-cold PBS and fixed with 70% ethanol for 2 h at −20 °C. The cells were subsequently washed with PBS and treated with 200 *μ*g/ml RNase and 500 *μ*l of PI (20 *μ*g/ml in stock) for 30 min in darkness at room temperature. PI-stained cells were assayed using BD FACSCalibur Flow Cytometry (Palo Alto, CA, USA) and cell cycle distributions (G0-G1, S, and G2-M) were analyzed with BD CellQuest Pro Software (built-in software; San Jose, CA, USA). Apoptosis was detected by staining the cells with an Annexin V-FITC Apoptosis Detection Kit from Strong Biotech Co. (Taipei, Taiwan) following the manufacturer's instructions. Five million HepG2 cells were stained for 15 min with Annexin V- FITC and PI at room temperature in darkness. After staining, the apoptotic cells were counted using BD FACSCalibur Flow Cytometry and the data were processed with the BD CellQuest Pro Software as described above.

### Determination of DNA fragmentation

HepG2 cells were pretreated with 20 *μ*M z-VAD-fmk or 50 *μ*M Nec-1 for 30 min, and then treated with 5 *μ*g Tan IIA for 12 h. The cells were collected and DNA was extracted using the Cell Genomic DNA Purification Kit (GeneMark, Taipei, Taiwan) according to the manufacturer’s protocol. DNA fragmentation was analyzed by 2% agarose gel electrophoresis, and stained with ethidium bromide.

### Cytotoxicity assays

HepG2 cells were pretreated with 20 μM z-VAD-fmk or 50 μM Nec-1 for 30 min, and treated with 5 or 10 *μ*g/ml Tan IIA for 1–36 h. LDH assay (Roche Co.) was performed as per manufacturer’s instructions. The absorbance was measured in a microplate reader (Thermo Scientific Multiskan Spectrum, New York, NY, USA) at 490 nm. Microsoft Excel 2010 was used for all data entry and analysis. Data were presented as mean±S.E. of the quadruplicate. Student's two-tailed *t*-test was used to analyze statistical significance between groups.

### Immunoprecipitation and western blot analysis

HepG2 cells were pretreated with 20 μM z-VAD-fmk or 50 μM Nec-1 for 30 min, and treated with 5 or 10 *μ*g/ml Tan IIA for 12 h. Cells were subsequently collected in 300 *μ*l of RIPA with protease inhibitor cocktail (Calbiochem) and homogenated. An aliquot (30 *μ*g) of the supernatant was retained for western blot analysis, and the remainder (500 *μ*g) was subjected to immunoprecipitation. Each supernatant was added with 1 *μ*g of the appropriate antibody and incubated for 24 h at 4 °C with rocking. An aliquot (10 *μ*l) of protein G agarose (Millipore, Temecula, CA, USA) was added to each sample and incubated for 2 h at 4 °C with rocking. Immunoprecipitates were collected by passing the samples through Cytosignal filters by centrifugation at 5000 r.p.m. for 5 min at 4 °C. The trapped beads were then washed four times with 500 *μ*l of lysis buffer (without Na3VO4, dithiothreitol, phenylmethylsulfonyl fluoride or protease inhibitor cocktail). An aliquot (100 *μ*l) of SDS–PAGE sample buffer was added to the trapped beads and the immunoprecipitated proteins were eluted by centrifugation at 5 000 g for 5 min. Samples were boiled for 5 min, resolved by SDS–PAGE, transferred to polyscreen membranes (PVDF; Millipore) and analyzed by immunoblotting using the appropriate antibodies (primary antibodies: cyclophilin A, HMGB1, caspase 8, caspase 3, RIP1, FLIP, RIP3, FADD (GeneTex, Irvin, CA, USA) and HRP secondary antibodies: anti-mouse and anti-rabbit, Abcam, Cambridge, UK). Blots were visualized by an HRP-conjugated substrate kit (BIO-RAD, Richmond, CA, USA), and the results were assayed using the Gel Documentation System.

### Statistical analysis

Data were presented as mean±S.D. Statistical analysis was based on one way Excel with a Student’s *t*-test; *P<0.05 was considered statistically significant.

## Abbreviations

Tan IIA: Tanshinone IIA
Nec-1: necrostatin-1
TNF: tumor necrosis factor
RIP1: receptor-interacting protein 1
RIP3: receptor-interacting protein 3
FLIP: FLICE inhibitory protein
MLKL: mixed-lineage kinase domain-like
TRAIL: (TNF)-related apoptosis-inducing ligand
FADD: Fas-associated death domain protein
LDH: lactate dehydrogenase
PVDF: polyvinylidene difluoride
PI: propidium iodide
pAb: polyclonal antibodies.

## Notes

Supplementary Informations

## References

[bib1] Vandenabeele P, Galluzzi L, Vanden Berghe T, Kroemer G. Molecular mechanisms of necroptosis: an ordered cellular explosion. Nat Rev Mol Cell Biol 2010; 11: 700–714. 20823910 10.1038/nrm2970

[bib2] Feoktistova M, Geserick P, Kellert B, Dimitrova DP, Langlais C, Hupe M et al. cIAPs block ripoptosome formation, a RIP1/caspase-8 containing intracellular cell death complex differentially regulated by cFLIP isoforms. Mol Cell 2011; 43: 449–463. 21737330 10.1016/j.molcel.2011.06.011 PMC3163271

[bib3] Micheau O. Cellular FLICE-inhibitory protein: an attractive therapeutic target? Expert Opin Ther Targets 2003; 7: 559–573. 12885274 10.1517/14728222.7.4.559 PMC2984612

[bib4] Safa AR, Pollok KE. Targeting the anti-apoptotic protein c-FLIP for cancer therapy. Cancers (Basel) 2011; 3: 1639–1671. 22348197 10.3390/cancers3021639 PMC3281420

[bib5] Imre G, Larisch S, Rajalingam K. Ripoptosome: a novel IAP-regulated cell death-signalling platform. J Mol Cell Biol 2011; 3: 324–326. 22114055 10.1093/jmcb/mjr034

[bib6] Tsuchiya Y, Nakabayashi O, Nakano H. FLIP the switch: regulation of apoptosis and necroptosis by cFLIP. Int J Mol Sci 2015; 16: 30321–30341. 26694384 10.3390/ijms161226232 PMC4691174

[bib7] Cai Z, Jitkaew S, Zhao J, Chiang HC, Choksi S, Liu J et al. Plasma membrane translocation of trimerized MLKL protein is required for TNF-induced necroptosis. Nat Cell Biol 2014; 16: 55–65. 24316671 10.1038/ncb2883 PMC8369836

[bib8] Chang TW, Lin CY, Tzeng YJ, Lur HS. Synergistic combinations of tanshinone IIA and trans-resveratrol toward cisplatin-comparable cytotoxicity in HepG2 human hepatocellular carcinoma cells. Anticancer Res 2014; 34: 5473–5480. 25275043

[bib9] Yu X, Deng Q, Li W, Xiao L, Luo X, Liu X et al. Neoalbaconol induces cell death through necroptosis by regulating RIPK-dependent autocrine TNFalpha and ROS production. Oncotarget 2015; 6: 1995–2008. 25575821 10.18632/oncotarget.3038 PMC4385831

[bib10] Han W, Xie J, Li L, Liu Z, Hu X. Necrostatin-1 reverts shikonin-induced necroptosis to apoptosis. Apoptosis 2009; 14: 674–686. 19288276 10.1007/s10495-009-0334-x

[bib11] Zhong ZH, Chen WG, Liu YH, Li QX, Qiu Y. Inhibition of cell growth and induction of apoptosis in human hepatoma cell line HepG2 by tanshione IIA. Zhong Nan Da Xue Xue Bao Yi Xue Ban 2007; 32: 99–103. 17344596

[bib12] Jeon YJ, Kim JS, Hwang GH, Wu Z, Han HJ, Park SH et al. Inhibition of cytochrome P450 2J2 by tanshinone IIA induces apoptotic cell death in hepatocellular carcinoma HepG2 cells. Eur J Pharmacol 2015; 764: 480–488. 26209360 10.1016/j.ejphar.2015.07.047

[bib13] Zhivotosky B, Orrenius S. Assessment of apoptosis and necrosis by DNA fragmentation and morphological criteria. Curr Protoc Cell Biol 2001; Chapter 18: Unit 18.13. 10.1002/0471143030.cb1803s12 18228342

[bib14] Chan FK, Moriwaki K, De Rosa MJ. Detection of necrosis by release of lactate dehydrogenase activity. Methods Mol Biol 2013; 979: 65–70. 23397389 10.1007/978-1-62703-290-2_7 PMC3763497

[bib15] Micheau O, Thome M, Schneider P, Holler N, Tschopp J, Nicholson DW et al. The long form of FLIP is an activator of caspase-8 at the Fas death-inducing signaling complex. J Biol Chem 2002; 277: 45162–45171. 12215447 10.1074/jbc.M206882200

[bib16] Boatright KM, Deis C, Denault JB, Sutherlin DP, Salvesen GS. Activation of caspases-8 and -10 by FLIP(L). Biochem J 2004; 382: 651–657. 15209560 10.1042/BJ20040809 PMC1133822

[bib17] Dohrman A, Russell JQ, Cuenin S, Fortner K, Tschopp J, Budd RC. Cellular FLIP long form augments caspase activity and death of T cells through heterodimerization with and activation of caspase-8. J Immunol 2005; 175: 311–318. 15972663 10.4049/jimmunol.175.1.311

[bib18] Yu JW, Jeffrey PD, Shi Y. Mechanism of procaspase-8 activation by c-FLIPL. Proc Natl Acad Sci USA 2009; 106: 8169–8174. 19416807 10.1073/pnas.0812453106 PMC2688887

[bib19] Pop C, Oberst A, Drag M, Van Raam BJ, Riedl SJ, Green DR et al. FLIP(L) induces caspase 8 activity in the absence of interdomain caspase 8 cleavage and alters substrate specificity. Biochem J 2011; 433: 447–457. 21235526 10.1042/BJ20101738 PMC4024219

[bib20] Lin Y, Devin A, Rodriguez Y, Liu ZG. Cleavage of the death domain kinase RIP by caspase-8 prompts TNF-induced apoptosis. Genes Dev 1999; 13: 2514–2526. 10521396 10.1101/gad.13.19.2514 PMC317073

[bib21] Feng S, Yang Y, Mei Y, Ma L, Zhu DE, Hoti N et al. Cleavage of RIP3 inactivates its caspase-independent apoptosis pathway by removal of kinase domain. Cell Signal 2007; 19: 2056–2067. 17644308 10.1016/j.cellsig.2007.05.016

[bib22] Moquin D, Chan FK. The molecular regulation of programmed necrotic cell injury. Trends Biochem Sci 2010; 35: 434–441. 20346680 10.1016/j.tibs.2010.03.001 PMC2904865

[bib23] Lee EW, Seo J, Jeong M, Lee S, Song J. The roles of FADD in extrinsic apoptosis and necroptosis. BMB Rep 2012; 45: 496–508. 23010170 10.5483/bmbrep.2012.45.9.186

[bib24] Ganten TM, Haas TL, Sykora J, Stahl H, Sprick MR, Fas SC et al. Enhanced caspase-8 recruitment to and activation at the DISC is critical for sensitisation of human hepatocellular carcinoma cells to TRAIL-induced apoptosis by chemotherapeutic drugs. Cell Death Differ 2004; 11: S86–S96. 15105837 10.1038/sj.cdd.4401437

[bib25] Vanden Berghe T, Linkermann A, Jouan-Lanhouet S, Walczak H, Vandenabeele P. Regulated necrosis: the expanding network of non-apoptotic cell death pathways. Nat Rev Mol Cell Biol 2014; 15: 135–147. 24452471 10.1038/nrm3737

[bib26] Wu YT, Tan HL, Huang Q, Sun XJ, Zhu X, Shen HM. zVAD-induced necroptosis in L929 cells depends on autocrine production of TNFalpha mediated by the PKC-MAPKs-AP-1 pathway. Cell Death Differ 2011; 18: 26–37. 20539307 10.1038/cdd.2010.72 PMC3131876

