# Supplementary material for: Simultaneous induction of apoptosis and necroptosis by Tanshinone IIA in human hepatocellular carcinoma HepG2 cells
Source: Cell Death Discov. 2016 Oct 3;2:16065–. doi: 10.1038/cddiscovery.2016.65 (PMC5045965; doi:10.1038/cddiscovery.2016.65)
Supplement: Supplementary Informations [file cddiscovery201665-s1.doc]

**Legend of supplementary information**

**S1. Expression levels of MLKL in trimer form were reduced by Tan IIA but recovered by co-treatment with both of z-VAD-fmk and Nec-1**

(a) Cell lysates were analyzed by Western blotting with the indicated antibodies. (b) Quantitative data are presented as mean ± S.D.; N=3 independent experiments. Statistical analysis was carried out using Student’s *t*-test (paired, one-tailed, **p* < 0.05, ** *p* < 0.01).


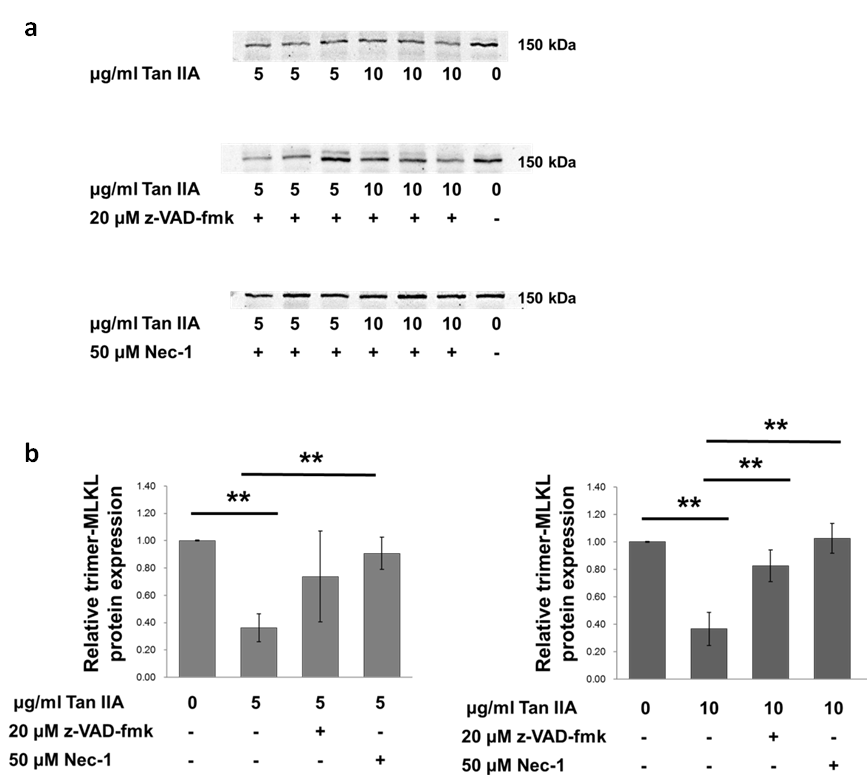


**S.1**
